# A synergistic negative effect of gestational smoke-exposure and small litter size on rat placental efficiency, vascularisation and angiogenic factors mRNA expression

**DOI:** 10.1371/journal.pone.0181348

**Published:** 2017-07-18

**Authors:** Zhen-Yan Chen, Ying Yao

**Affiliations:** Department of Nutrition, Tongji Hospital, Tongji Medical College, Huazhong University Science and Technology, Wuhan, Hubei, China; University of Southampton, UNITED KINGDOM

## Abstract

Smoking increases the risk of pregnancy complications such as spontaneous abortion and low birth weight (LBW). By cigarette smoke exposure (gestational day, GD3-17), normal-litter-size pregnancy with low birth weight (NP-LBW) and small-litter-size pregnancy with normal birth weight (SP-NBW) in rats were induced. The placental weight in SP-NBW was twice the weight of the normal in contrast with the smaller placenta in NP-LBW. Compared with the normal, placental efficiency (expressed as fetus-to-placenta weight ratio) and placental vascularisation were significantly decreased in smoke exposed placentas with more obvious decrease in SP-NBW. For NP-LBW, decreased placental vascularisation was due to decreased labyrinth vascularisation which was caused by both decreased number density and diameter of fetal capillary. For SP-NBW, decreased placental vascularisation was due to reduced proportion of labyrinth in placenta and decreased labyrinth vascularisation which was caused by decreased fetal capillary number density. Real time RT-PCR analysis showed a tendency for decreased placental mRNA level of vascular endothelial growth factor (VEGF), angiopoietin-1 (Ang1) and tyrosine kinase receptor-2 (Tie2) in NP-LBW(*P*<0.1), and the tendency became obvious in SP-NBW(*P*<0.05). A tendency for decreased placental mRNA level of fms-like tyrosine kinase-1(Flt1) and angiopoietin-2 (Ang2) was also observed in SP-LBW(*P*<0.1). Our data demonstrated the synergistic negative effect of gestational smoke-exposure and small litter size on placental efficiency, placental vascularisation and placental angiogenic growth factor mRNA expression in rat.

## Introduction

Smoking during pregnancy is associated with adverse pregnancy outcomes including spontaneous pregnancy loss and low birth weight. It has been reported that smoke exposure during pregnancy increased the risk of miscarriage by nearly 11% and reduced birth weight by nearly 10.75% [1]. Placental vascularisation is essential for normal pregnancy and determines the transport of oxygen and nutrients from the maternal blood circulation to the fetal blood circulation. Impaired placental vascularisation is thought to be associated with adverse pregnancy outcomes. However, findings on the effects of maternal smoking on the structure of human placental vasculature are inconsistent [2–10]. Several studies had demonstrated reduced placental vascularisation in smokers [2,3,7,8,9] in marked contrast to the adaptive changes seen in placenta associated with other types of preplacental hypoxia [11], while others used scanning electron microscopy on casts of the capillaries or by 3D microscopy observed an adaptive response of the villous capillaries in placentas of heavy smokers with increased branching [6,10]. Animal model of fetal growth restriction triggered by constituents of cigarette smoke revealed extensive branching and enlargement of vessels within the small placental labyrinth was also observed [12]. Vascularisation is controlled by angiogenic factors. Vascular endothelial growth factor (VEGF) and its receptors, fms-like tyrosine kinase-1(Flt1, also named VEGFR-1) and fetal liver kinase-1 (Flk1, also named VEGFR-2) are essential for vasculogenesis. Angiopoietin-1(Ang1), Angiopoietin-2(Ang2), and their receptor (Tie2) are essential for vascular remodeling. VEGF, Flt1 and Flk1 are essential for endothelial cell proliferation, migration and tube-like structure formation, while Ang1 promotes the association of endothelial cells with periendothelial cells to mature and stabilize newly formed blood vessels. Ang2 loosens the vessel wall, rendering endothelial cells accessible to VEGF to further promote angiogenesis [13]. So far, studies investigated the alterations of VEGF/VEGFR and Ang/Tie2 expression on placenta from maternal smoking are seldom and limited to VEGF and VEGFR-1 on the assumption that maternal smoking is associated with a decreased risk of preeclampsia through reduced soluble form of VEGFR-1 which acts as a negative regulator of VEGF [14]. However, the results are inconsistent. Some study showed no changed VEGF and VEGF receptor 1 mRNA in human placenta of smoking mothers of first trimester [15,16], while increased placental VEGF mRNA [17] and protein [18] of smokers of first trimester, and decreased soluble VEGF receptor 1 [14] were also reported.

Placental efficiency (fetus-to-placenta weight ratio) was positively correlated with placental vessel density and placental VEGF mRNA as all increased progressively as gestation proceeds [19–21]. However, comparative studies failed to demonstrate this positive relationship between different placental efficiency groups of the same gestational stage [20–22]. In contrast with the consistent finding of reduced birth weight by passive smoking, placental weight was reported to be decreased [5], increased [6, 10,23] and no change with cigarette smoke [2,3,4,7,8]. In many of these studies, placental efficiency was smaller in smokers than in nonsmokers, but this conclusion has not been established. Distinct individual difference of placental efficiency existed even in the same litter. So the aim of this study was to determine the alterations of placental efficiency, placental vascularisation and angiogenic factors expression in placenta with smoke exposure and the interaction between them.

## Materials and methods

### Modeling

Specific pathogen free grade, Sprague Dawley rats were purchased from Hunan Slac Jingda Laboratory Animal Co., Ltd. (China). Animals were housed and maintained in the Laboratory Animal Care center of Tongji Medical College and kept in a controlled environment at a temperature of 24 ± 3°C, a humidity of 50 ±10% and a 12-h light/12-h dark cycle, and given free access to a standard diet and distilled water. Female rats aged between 8–12 wk with sperms present in vaginal smears following mating (designated as gestation day, GD 0) were subjected to cigarette smoke in a chamber measuring 50 cm×28 cm×20 cm (4 rats per chamber) for five periods of 30 min, with 1.5 hour interval between exposures from gestation day 3 till gestation day 17 in model group (five cigarettes lit for each exposure, the tar and nicotine yields of commercial cigarettes were 15 and 1.2 mg per cigarette respectively), non-smoke-exposed normal pregnant rats served as controls. All animal experiments were performed according to the institutional guidelines established by the Animal Care and Use Committee of Tongji Medical College, Huazhong University of Science and Technology with Permit Number: TJ-A20130901.

### Tissue collection

On GD17, pregnant rats were anesthetized with pentobarbital (dosage of anesthetics was 50mg/kg), each feto-placental unit was removed quickly from the uterus, and the fetuses and placentas were weighed. Three placentas randomly selected from each pregnancy were used for analysis as one sample and then the dams were euthanized by cervical dislocation. Before further processing, placenta was placed flat, rotated at random, bisected with a vertical cut through the insertion of the umbilical cord to expose a cross-section of the labyrinth and non-labyrinth areas which paralleled to the mesometrial-fetal axis, and then was halved. The three halves of the placentas were homogenized and preserved at -80°C for RNA extraction as one sample, while the other halves were fixed with 4% polyoxymethylene and then embedded in paraffin with the cutting area faced to a surface of paraffin block ensuring standardization of sections. Tissues were then sectioned at 4μm for fetal capillaries staining.

### Fetal capillaries staining and image analysis

Rat placenta consists of three morphologically distinct zones, decidua, junction and labyrinth. Junction zone is the major secreting zone with no fetal capillary located in it. Labyrinth, major site of nutrient and gas exchange, is composed of fetal capillaries and maternal blood sinusoids. Fixed tissue sections were immunostained with biotinylated lectin BS-1(cat #L3759, Sigma, UAS) which could specifically combined with vascular endothelial cells to identify fetal capillaries in labyrinth as described in our previous work [19]. Three placentas per pregnancy with one section per placenta were used for analysis. Images were taken by the Canon Micro-imaging System (Canon 350 D, Japan) and analyzed by Image-Pro Plus version 6.0 (Media Cybernetics, Silver Spring, MD, USA) incorporated with a scale for measurement. To determine proportion of labyrinth in placenta (proportion of placental sectional area that was occupied by labyrinth), non-overlapping fields of view with each placental section being fully covered were captured at 4×(objective) magnification. Running the Grid mask program of the Image-Pro Plus software, an 111-point grid superimposed on each field of the section, proportion of labyrinth in placenta was then calculated by dividing the total numbers of points falling on labyrinth by the total numbers of points falling on placenta. Labyrinth was studied in more detail at 40×(objective) magnification to determinate the maternal blood sinusoids (MBS) area density (proportion of labyrinth sectional area that was occupied by maternal blood sinusoids), fetal capillaries (FC) area density, FC number density and FC diameter in labyrinth. Observed the hierarchical character of labyrinth, 1 to 2 fields each from the center (chorionic plate side) and the periphery (junction zone side) were selected per placenta, yielding a total of 5–6 fields for the center and the periphery within labyrinth each per pregnancy. MBS area density was calculated by dividing the total numbers of points falling on maternal blood sinusoids area in the 5–6 fields by the total numbers of points falling on labyrinth in the 5–6 fields. FC area density was calculated in the same way. FC number density in labyrinth was calculated by dividing the total numbers of fetal capillary in the 5 fields by the total area of labyrinth of the 5–6 fields (mm2). FC diameter meant the shortest distance that was perpendicular to the longest aspect of vessel and all fetal capillaries in the selected fields were measured.

### Realtime RT-PCR

VEGF, Flt1, Flk1, Ang1, Ang2, and Tie2 mRNAs levels were analyzed by the quantitative RT-PCR method. All reagents for the quantitative RT-PCR were purchased from TaKaRa (Japan). Total RNA was extracted using Trizol reagent. RNA purity and concentration were measured using a nucleic acid/protein analyzer (Beckman Coulte DU730, USA). One microgram of RNA was reverse transcribed using a PrimeScript RT Reagent Kit with gDNA Eraser. The volume of the reaction mixture for mRNA amplification was 20μL and contained 2 μL of transcribed cDNA, 0.4 μL forward primer (10 μmol/L), 0.4 μL reverse primer (10 μmol/L), 0.4 μL passive reference dye, 7.8 μL sterile water and 10 μL 2×SYBR Premix Ex Taq. Reactions were performed in 48-well optical PCR plates using an Applied Biosystems StepOne Real-Time PCR System (Applied Biosystems, USA). Each sample was assayed in triplicate. The primer sequences for VEGF, Flt1, Flk1, Ang1, Ang2, and Tie2 mRNA were detailed in Table 1. All data were normalized to GDPAH, and the expression of one placental in normal group was assumed to be 1 and used as a reference. 2^-ΔΔCT^ method was used to calculate the relative amounts of the target genes.

**Table 1:** Primers used for real-time PCR.

Genes	Forward primers	Reverse primers
VEGF	GTCCTCACTTGGATCCCGACA	CCTGGCAGGCAAACAGACTTC
Flt1	CGG TTT GCT GAA CTT GTGGAG A	GGG ACT GAG TAT GTG AAG CCA CTG
Flk1	AATGCCCATGACCAAGAATGT	GGATAGAGCCGCGTGTCTGAA
Ang-1	CACCGTGAGGATGGAAGCCTA	TTCCCAAGCCAATATTCACCAGA
Ang-2	CAGTAGCATCAGCCAACCAGGA	GACCACATGCTGCGAACCAC
Tie2	GATGGACGCTGCCATCAAGA	TGATGTTCGGATGGTGTCCAA
GDPAH	GGCACAGTCAAGGCTGAGAATG	ATGGTGGTGAAGACGCCAGTA

### Statistical analysis

Categorical variables were expressed as percentage and continuous variables were presented as mean±standard deviation. General linear model with smoke exposure and litter size (normal or small, small defined as<5) as fixed effects was used to test their effects on fetal weight, placental weight, placental efficiency and placental vascular variables. Test batch was added as another main effect in angiogenic factors mRNA analysis of general linear model. All data analyses were performed using SPSS 17.0. *P*<0.05 was considered statistically significant and *P*<0.1 a trend.

## Results

### Effects of smoke exposure on litter size, birth weight, placental weight and placental efficiency

Litter size was an index of a combination of embryo implantation and abortion, while birth weight was an index of fetal growth. In the smoking-exposed group, 17% (5/29) pregnant rats had the fetuses be completely aborted, 17% (5/29) pregnant rats had small litter size (3 rats gestating single fetus and 2 rats gestating 3 to 4 fetuses). Neither completely aborted pregnancy nor small-litter-size pregnancy was observed in normal group. By a mixed effects analysis of variance, smoke exposure had a negative effect on fetal weight (F = 138.5, *P* = 0.000), placental weight (F = 11.2, *P* = 0.001) and placental efficiency (F = 45.9, *P* = 0.001). Smoke exposure yielded 18% reduction in fetal weight, 6.8% reduction in placental weight and 15% reduction in placental efficiency. As expected, small litter size had a positive effect on fetal weight (F = 8.6, *P* = 0.004), placental weight (F = 456.5, *P* = 0.000), and a synergistic negative effect (F = 42.1, *P* = 0.001) with smoke exposure on placental efficiency. Placental weight was increased by 111% and placental efficiency was decreased by 56% in small litters compared to the corresponding normal pregnancy. Detailed results of litter size, birth weight, placental weight and placental efficiency were present in Table 2.

**Table 2:** Comparisons of litter size, fetal weight, placental weight and placental efficiency.

group	Litters	N litters	Litter size	Fetal weight(g)	Placental weight(g)	placental efficiency
Control	Normal litters	8	13±2	1.19±0.23	0.44±0.87	2.80±0.85
Smoke- exposed	Normal litters	19	13±3	0.97±0.11#	0.41±0.06#	2.37±0.35#
	Small litters	5	2±1#	1.12±0.23	0.92±0.17#	1.24±0.16#

Values are the mean±SME,

^#^*P*<0.05, *vs* control group. Given no decreased birth weight in small litters, smoke-exposed pregnancy was then subdivided into small-litter-size pregnancy with normal birth weight (SP-NBW) and normal-litter-size pregnancy with low birth weight (NP-LBW) (no fetal dead was observed in current study, absorbed conceptus was excluded in litter size counting).

### Effects of smoke exposure and litter size on proportion of labyrinth in placenta and labyrinth vascularisation

**Fig 1:**
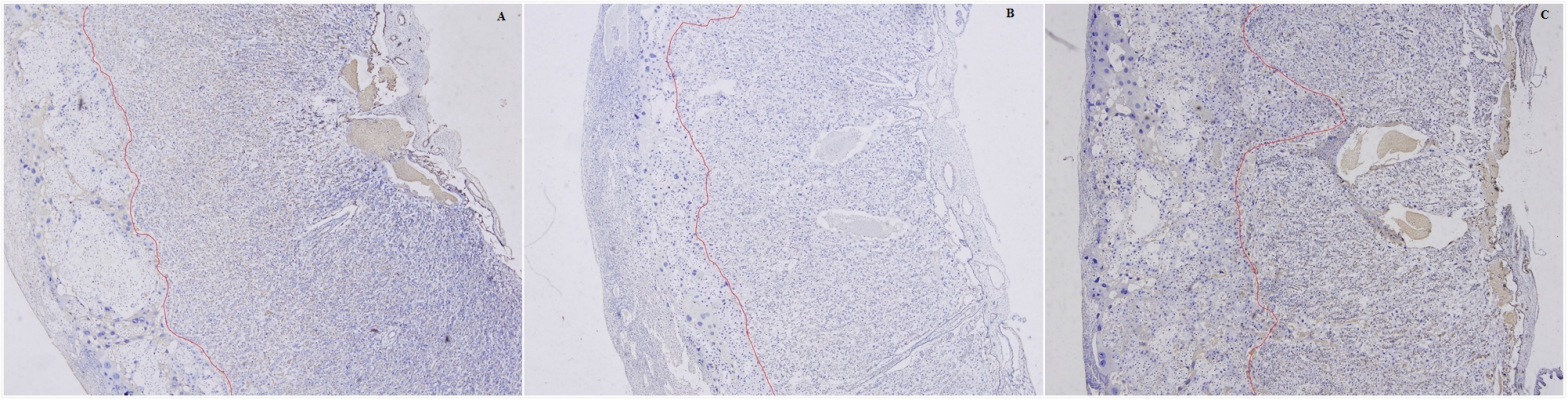
Comparison of proportion of labyrinth in placenta between the groups. The figure showed mid-line sections of placentas on GD17 that were subjected to hematoxylin staining with magnification 4x (objective) from normal group (A), NP-LBW group (B) and SP-NBW group (C). Placental tissue on the right side of red boundary line represented labyrinth. Note that proportion of labyrinth in placenta was reduced in SP-NBW than that in normal placenta.

**Fig 2:**
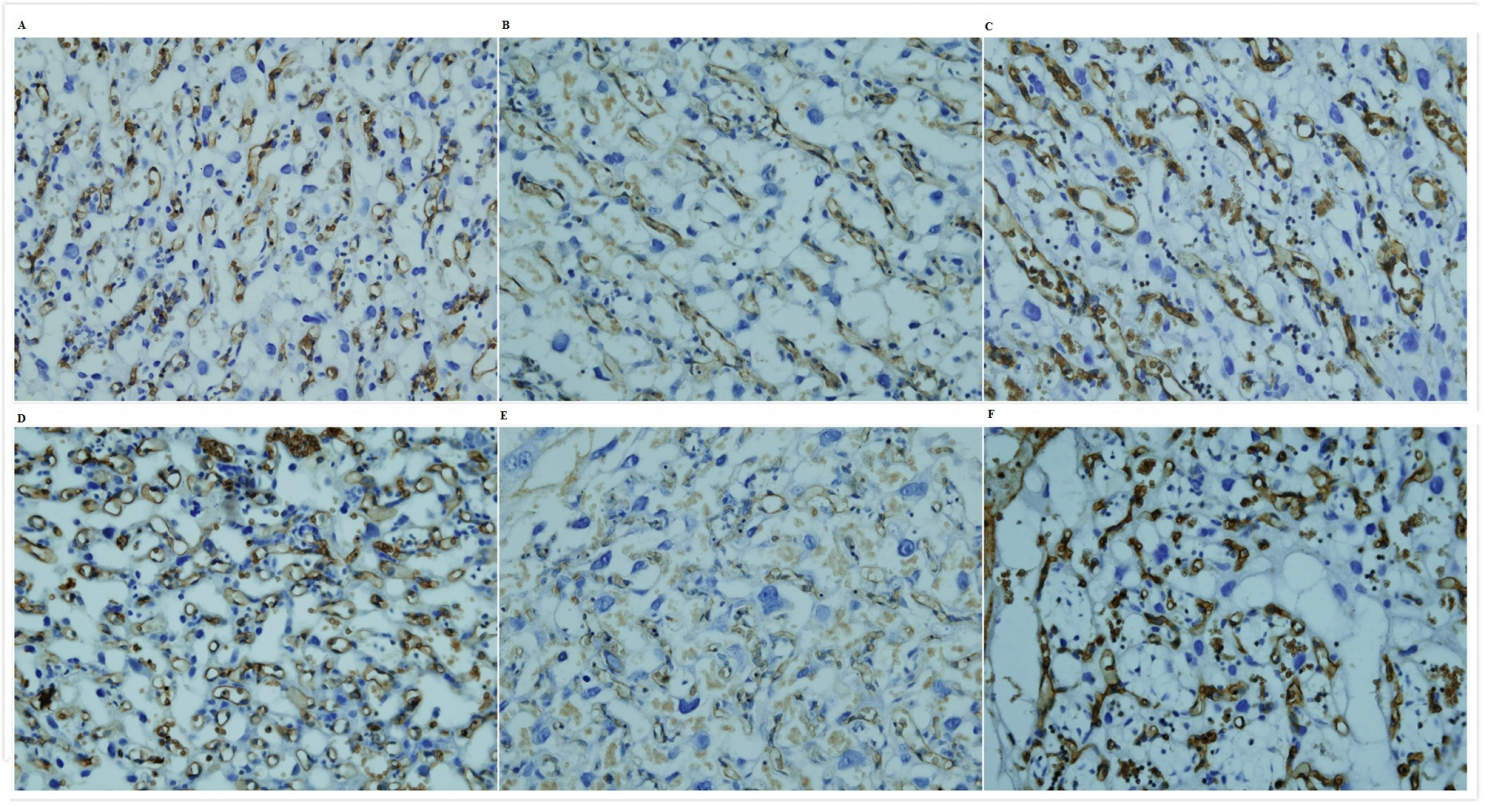
Comparison of fetal capillary number density, area density, diameter, and maternal blood sinusoids area density between the groups. The figure showed mid-line sections of labyrinths on GD17 that were subjected to histochemistry staining by BS-1 lectin with magnification 40x (objective) from normal group (A, **D**), NP-LBW group (B, **E**) and SP-NBW group (C, **F**). A, B, C were from central part of labyrinth and D, E, F were from the periphery of labyrinth. Clay bank rings represent fetal capillaries.

Image analysis revealed that small litter size (F = 30.2, P = 0.000) but not smoke exposure (F = 0.9, P = 0.342) reduced proportion of labyrinth in placenta. Reduced proportion of labyrinth in placenta by 21% was only observed in SP-NBW in compared with normal placentas (Fig 1). Based on the immunohistochemical staining with BS-1lectin, images of maternal blood sinusoids and fetal capillaries in labyrinth were obtained. Image analysis revealed hierarchical structure of placental vertical section. Fetal capillary number density, area density, diameter, and maternal blood sinusoids area density in the periphery were approximately 115.5%, 61.2%, 79.4% and 135.7% of those in the center of labyrinth in normal placentas respectively (*P*<0.05). For fetal capillaries number density, smoke exposure and small litter size had synergistic negative effects on it, leading to more obviously reduced fetal capillaries number density in SP-NBW placentas. For fetal capillary diameter, small litter size had enlarged effect in contrast with the constricting effect of smoke exposure. Consequently, fetal capillaries area density was equally decreased and maternal blood sinusoids area density was equally increased in SP-NBW and NP-LBW. For SP-NBW, decreased placental vascularisation was due to reduced proportion of labyrinth in placenta and decreased labyrinth vascularisation which was caused by decreased fetal capillary number density. For NP-LBW, decreased placental vascularisation was due to decreased labyrinth vascularisation which was caused by both decreased number density and diameter of fetal capillary (Table 3, Fig 2).

**Table 3:** Comparisons of proportion of labyrinth in placenta and vascularisation of the labyrinth.

	Control(n = 8)	Smoke- exposed	
		NP-LBW(n = 11)	SP-NBW(n = 5)
Proportion of labyrinth in placenta	0.684±0.066	0.666±0.031	0.536±0.039^c^
Central FC number density (n/mm^2^)	246±40	156±46^a^	112±16^c^^e^
Peripheral FC number density(n/mm^2^)	285±13	180±33 ^a^	145±8^c^^e^
Central FC diameter (um)	18.8±5.8	17.9±5.7^a^	22.4±8.6^c^
Peripheral FC diameter (um)	14.7±4.9	15.7±6.2^a^	15.7±4.9^c^
Central FC area density	0.067±0.166	0.037±0.021^a^	0.040±0.015^c^
Peripheral FC area density	0.041±0.013	0.027±0.009^a^	0.028±0.005^c^
Central MBS area density	0.140±0.522	0.211±0.064^a^	0.238±0.052^c^
Peripheral MBS area density	0.190±0.066	0.257±0.548^a^	0.275±0.044^c^

Definition of abbreviations: FC = fetal capillary; MBS = maternal blood sinusoids. Values are the mean ±SME,

^a^, *P*<0.05, effect of smoke exposure;

^c^, *P*<0.05, effect of small litter size;

^e^, *P*<0.05, synergistic effect of smoke exposure and small litter size.

### Effects of smoke exposure and litter size on placental angiogenic factors mRNA expression

By a mixed effects analysis of variance, these were trends of decreased VEGF mRNA by 25%, Ang1 mRNA by 43%, and Tie2 mRNA by 55% with smoke exposure (*P*<0.1). When combined with small litter size, significantly decreased VEGF mRNA by 60%, Ang1 mRNA by 71%, and Tie2 mRNA by 70% were observed (*P*<0.05). The synergistic effect of smoke exposure and small litter size lead to trends of decreased Flt1 mRNA by 60% and decreased Ang2 mRNA by 71% (*P*<0.1). The expression level of Flk1 did not get significant difference between the groups (Table 4).

**Table 4:** Comparison of relative placental VEGF-A, Flt1, Flk1, Ang1, Ang2, and Tie2 mRNA expression.

mRNA	Control(n = 8)	Smoke- exposed	
		NP-LBW(n = 19)	SP-NBW(n = 5)
VEGF	1.50±0.49	1.12±0.38^b^	0.58±0.23^c^^e^
Flt1	1.41±0.55	0.88±0.67	0.57±0.05^f^
Flk1	0.819±0.448	0.603±1.89	0.082±0.064
Ang1	1.547±1.570	0.874±0.440 ^b^	0.552±0.269 ^d^
Ang2	1.46±1.07	0.845±0.395	0.418±0.175^f^
Tie2	1.141±0.328	0.675±0.481^b^	0.339±0.088^c^^d^

Values are the mean±SME.

^b^, *P*<0.1, effect of smoke exposure;

^c^, *P*<0.05, effect of small litter size;

^d^, *P*<0.1, effect of small litter size;

^e^, *P*<0.05, synergistic effect of smoke exposure and small litter size.

^f^, *P*<0.1, synergistic effect of smoke exposure and small litter size.

## Discussion

Beside intrauterine growth restriction which is the best documented among the variety of adverse outcomes by cigarette smoke exposure in utero, human epidemiological study revealed that tobacco use was independently associated with an increased risk of spontaneous abortion (odds ratio 1.8) [24] and secondhand tobacco smoke exposure was associated with increased risk of failed implantation and reduced IVF success (odds ratio 1.5) [25]. Gestational day 6 is the peri-implantation stage in rat. In theory, smoke exposure initiated on GD3 is possible to induced abortion. However, animal model of cigarette smoke-induced abortion was rare reported and effect of peri-implantation smoke exposure on litter size was not found in previous studies [26–28]. The negative results might due to the low occurrence rate of smoke induced abortion. Compensatory mechanisms are sufficiently robust to resist the damage of smoking and allow pregnancy to continue, although placental function is impaired, and ultimately, lead to intrauterine growth restriction. If the damage intensified and outstrips potential repair mechanisms, abortion will occur. The insult might be not intense enough for abortion induction in previous studies. Emily R et al exposed pregnant mice to cigarette smoke during pre-/peri-implantation (GD1–5), throughout gestation (GD 1–17) and post-implantation (GD6–18) [26]. He found no effect of cigarette smoke on litter size on GD18, and smoke exposure during pre-/peri-implantation (GD1–5) or throughout gestation (GD 1–17) resulted in low birth weight whereas smoke exposure during post-implantation (GD6–18) did not [26]. However, our preliminary experiment found that low birth weight could be successfully induced by post-implantation smoke when the insult intensified. In Emily R et al study, smoke exposure (GD1-17) reduced birth weight by 6.1% whereas reduction was 18% in this study which might indicate more intensified insult in our study. However, abortion seemed to be all-or-none with smoke exposure in this study. Inter-individual difference in sensibility to the toxic substances might contribute to the different outcomes.

Reduced placental weight and efficacy in maternal smoking was reported in 2014 by R.H.F. van Oppenraaij et al who conducted a large human epidemiological sample study (n = 7945) with multivariable linear regression models adjusting for potential confounder [29]. Our study firstly demonstrated reduced placental efficacy with smoke exposure in animal model. Inconsistent reports before would caused by small multitude of the placental weight change that could be easily confounded by other factors when the insult is not intense enough. Histomorphometric studies on human placentas of smokers show contrasting effects of smoking on placental vascularisation [2–11]. Beside variance of preparations, sampling and analysis that contribute to the inconsistent results, compensatory response related to the start time, duration and magnitude of insult, and individual difference also affects the results. Our study examined placenta of late-gestation while the previous studied term (when placenta was degenerated) [2,3,4,6,7,8] or first trimester placenta (would before the onset of vascular density reduction) [5,9,10]. Compensatory response would have occurred in previous studies that reported increased villous capillaries. Unlike other types of preplacental hypoxia (maternal anemia or pregnancies at high altitude) which adaptive changes with increased capillary volume fractions are confirmed [11], decreased capillary volume fraction in cigarettes smoke during pregnancy was reported [2,3,7,8,9], indicated that other influences suppress the compensatory response (eg, a toxic effect).

Except increased placental VEGF mRNA [17] and protein [18] were reported, alterations of VEGF system and angiopoietin system gene expression levels in smoking pregnant women placenta were seldom reported. Akihiko Sekizawa et al reported increased placental VEGF mRNA in first trimester placentas of smokers [17], however, when number of samples increased, no difference was found [15]. Previously, we found decreased Tie2/Ang1 in smoke exposed rat placentas of GD17 [19]. We increased the number of samples in this study and still found decreased trend of VEGF/tie2/Ang1 in smoke exposed placentas. Trophoblast cells respond to reduced oxygen tensions by activation of the VEGF system and stimulate angiogenesis [30], cigarette smoke extracts was demonstrated to inhibit hypoxia-induced angiogenesis of human umbilical vein endothelial cells via reduced expression of HIF-1 and VEGF in hypoxic conditions [31]. VEGF are up-regulated in several inflammatory conditions, smoking can cause inflammation [32]. We hypothesized that the inconsistent results might be caused by the contrasting effect of hypoxia, inflammation and toxic effect of ingredients of tobacco. Compensatory response would be determined by the combined effect of the contrasting factors. Except one pregnancy, compensatory response was not observed with smoke exposure and seemed to be all-or-none in this study. Placental efficacy and vessel density decreased in smoke exposed in association with decreased placenta angiogenic factors mRNA. The consistent change supports the hypothesis that smoking causes anti-angiogenic status and leads to reduced placental angiogenic factors mRNA expression, placental vascularisation is reduced, and ultimately, reduced placental efficiency.

Kimberly A et al failed to demonstrate the hypothesis that with an increase of fetal number within the uterus placental capillary vascularity will be enhanced to compensate for reduced placental weight per fetus, and the expression of angiogenic factors in the placenta will depend on the number of fetuses [20–22]. In this study, we confirmed this hypothesis by the consistenly decreased placental efficiency, vascularity and angiogenic factors mRNA in SP-NBW. Large litter size difference of ours might contribute to the positive results.

To our knowledge, this study is the first to provide consistent changes of placental efficiency, placental vascularisation and placental angiogenic factors mRNA expression in smoke-exposed placenta. Decreased fetal capillary number density in smoke-exposed placenta was also firstly presented. Only one previous study reported reduced total villous capillaries number in smokers [8], other studies concluded that reduced capillary volume density was only due to capillaries diameter reduction [2,3,7,9]. The strengths of our study include standardization of the orientation of placental section and the fields in center and periphery separated selected. Placental vertical cross-section showed that placenta vasculature was a tree-like structure where stem-vasculature lies in the center and microvasculature lies in the periphery. Significant differences in vascular morphology exist between stem vasculature and microvasculature, so our study was one of the few studies which selected central and peripheral fields separately to better elucidate the alterations. The limitation in our study is that we did not evaluate the expression of VEGF family proteins which would have been beneficial in correlating with the mRNA levels and detect the serum nicotine level to better explain the adverse outcomes. In SP-NBW, reduced placental vascularisation was due to the reduced proportion of labyrinth in placenta and decreased labyrinth vascularisation which only caused by decreased fetal capillary number density. In NP-LBW, reduced placental vascularisation was only due to decreased labyrinth vascularisation which caused by both decreased number density and diameter of fetal capillary. We could not explain the mechanism of the different placental vascular changes between SP-NBW and NP-LBW. We also could not elucidate the exact mechanism of angiogenic factors mRNA alteration by smoke exposure. Further studies are needed to elucidate these mechanisms.

## Supporting information

S1 Datesets Original data for the results of this experiment. Data for litter size, birth weight, placental weight, placental efficiency, proportion of labyrinth in placenta, labyrinth vascularisation, fetal capillary diameter and placental angiogenic factors mRNA expression.
(XLS)
